# Current Estimates for HIV-1 Production Imply Rapid Viral Clearance in Lymphoid Tissues

**DOI:** 10.1371/journal.pcbi.1000906

**Published:** 2010-09-02

**Authors:** Rob J. De Boer, Ruy M. Ribeiro, Alan S. Perelson

**Affiliations:** 1 Theoretical Biology and Bioinformatics, Utrecht University, Utrecht, The Netherlands; 2 Theoretical Biology and Biophysics, Los Alamos National Laboratory, Los Alamos, New Mexico, United States of America; Imperial College London, United Kingdom

## Abstract

It has recently been estimated that a single HIV-1 infected cell produces between 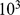 and more than 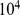 viral particles over its life span. Since body-wide estimates of the ratio of free virus to productively infected cells are smaller than 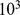 and much smaller than 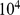, individual virions must be cleared rapidly. This seems difficult to reconcile with the fact that most of the total body virus is trapped on follicular dendritic cells where it can survive for many months. It has also been difficult to reconcile the vast difference in the rates at which the virus is cleared from the blood in rhesus macaques and in chronically infected patients. Here we attempt to reconcile these seemingly contradictory observations by considering the virion clearance rate in various organs and the virion exchange rates between them. The main results are that the *per capita* clearance rate of free virus in lymphoid tissue should be fast, the virion exchange rate between lymphoid tissue and the blood should be slow, and the comparatively slow previous estimates for the virion clearance rate from the blood correspond to the rate of virion efflux from the blood to other organs where the virus is ultimately cleared.

## Author Summary

A human cell that is infected with the AIDS virus HIV-1 may produce more than  new viral particles over its short life span. In patients chronically infected with HIV-1, one can estimate that on average there are much less than  free viral particles per productively infected cell. This suggests that the rate at which individual virus particles are cleared from the body must be fast. Most of the virus is long-lived, however, because it is trapped on follicular dendritic cells. We attempt to reconcile these seemingly contradictory observations by estimating the virion clearance rate in various organs, and the virion exchange rates between them, using a mathematical modeling approach. We find that individual virus particles are cleared rapidly from the lymphoid tissue, and that the rate at which virus is exchanged between lymphoid tissue and the blood is slow.

## Introduction

The major targets of HIV and simian immunodeficiency virus (SIV) infection are CD4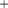 T cells [1]. During the acute stage of infection large numbers of resting and memory CD4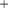 T cells disappear from the lymphoid tissues and mucosal layers, particularly in the gut, by direct infection and by bystander effects. Lymphoid tissue remains the primary site of infection after acute infection has resolved and the viral load approaches a steady state called the “set-point” [1]. Chronic infection is characterized by a set-point viral load, and rapid turnover of productively infected cells [2]–[4]. To maintain this steady state requires a balance between virus production and clearance, and between target cell production and death. Combining image analysis with *in situ* hybridization in lymphoid tissue from patients chronically infected with HIV-1, the total number of productively infected CD4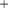 T cells has been estimated to be 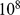 cells, and the total number of HIV-1 particles has been estimated to exceed 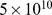 virions [5], [6]. At steady state, one could naively conclude that a single productively infected CD4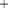 T cell should therefore account for a viral load of approximately 500 virions. We shall show below that the situation is more complex.

To understand viral production and clearance better, one needs to consider the current quantitaive estimates of viral production and clearance, as well as where these processes are occurring in the body. Most of the total body virus is located in lymphoid tissues, typically in association with follicular dendritic cells (FDCs) [1], [5]. FDCs trap virus and retain it on their surface for many months [7]–[11]. The FDC associated virus pool fills up during early infection, i.e., not later than a few days after onset of symptoms, and does not expand over the course of chronic HIV-1 infection [12]. Although most of the virus resides in this fairly constant storage on FDCs, the store rapidly declines during antiretroviral treatment (ARV) [13], suggesting the existence of a quasi steady state between free and FDC associated virus in the lymphoid tissue [8]–[10], [14]. In a mouse model it was shown that a small fraction of HIV-1 persisted on FDCs, and remained infectious over a period of 9 months [7]. A recent study confirmed the long-lived nature of this reservoir in humans [11].

Viral clearance rates have been estimated in the blood, and different techniques have yielded a variety of estimates [4], [15]–[17]. Rapid clearance rates with half-lives of 3–4 minutes were found after infusion of SIV into the blood of uninfected and infected rhesus macaques [16], [17]. In patients chronically infected with HIV-1, more than 10-fold slower clearance rates were found using plasma apheresis to increase viral clearance, and hence perturb the viral set point [15]. By plasma apheresis approximately 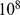 particles were removed over a period of two hours, and this reduced the viral load in blood, with a nadir that was approximately half of the original viral load [15]. The mere fact that the removal of less than 1% of the total body virus lead to an observable decline in the plasma virus load [15], suggests that the exchange of virus between the lymphoid tissues and the blood cannot be rapid [14]. Further, sequence analysis of virus in splenic white pulps suggests that virus trapped on FDC is produced locally [18], supporting the notion of slow viral exchange between blood and lymphoid tissue.

Virus production rates have also been estimated by several techniques. Because in other lentivirus infections, most notably visna virus, intracellular viral DNA levels increase approximately exponentially and then virus appears to be released rapidly [19], the term burst size is commonly used to describe the total amount of virus produced by an infected cell [20]. If one knows the burst size, 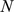, and the average lifespan of a productively infected cell, 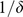, then the viral production rate, 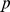, is given by 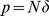. Note that 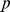 is then the average rate of virion production over the lifespan of a productively infected cell. Current estimates suggest 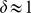 d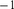 [21]. Thus, the burst size corresponds to the daily viral production rate.

Recent studies have combined image analysis with *in situ* hybridization to estimate burst size. Assuming that the maximal HIV RNA count in a cell corresponds to the burst size, Haase *et al.* [5] estimated a production rate of approximately a hundred particles over the life span of a productively infected cell. Hockett *et al.* [22] quantified more precisely the amount of viral RNA (vRNA) per cell by a PCR technique. They found an average of 3900 (range 3162–5011) vRNA copies per infected cell, and because of limited variation in the number of copies per cell, they concluded that viral production is a few thousand virions per cell, and also assumed that bursting was an all-or-none phenomenon [22]. However, the estimates by Haase *et al.* [5] and Hockett *et al.* [22] are based on measuring HIV RNA at a single time point. If infected cells continue to produce virus over an extended period, then these estimates would be underestimates of the true total cellular production of virus.

One can also attempt to measure burst size by directly imaging the extracellular viral particles surrounding an infected cell [23]. Using this method, Reilly *et al.* [23] found 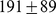 and 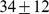 copies of HIV-1 RNA surrounding infected activated and resting CD4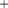 T cells, respectively, in the lymphoid tissue of acutely SIV-infected rhesus macaques. They then fitted a five parameter model to this data, with three of the parameters describing the rate of viral production as a function of time since infection (see Methods), and the remaining parameters describing the rate of exponential decay of cells producing virus, and the rate of loss of viral particles. Using this model, they estimated that the half-life of virus located around CD4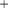 T cells producing virus in lymphoid tissue was approximately three hours. However, this half-life combined diffusion of virus out of the local area and true virion clearance [23]. Even if all loss was due to clearance, a three hour half-life corresponds to a per virion clearance rate of 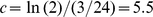 d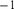. With this estimate, Reilly *et al.* [23] calculated median production rates of approximately 1500 and 1400 viral particles per activated cell, and of approximately 650 and 3400 viral particles per resting cell, depending on two different assumptions for the half life of productively infected resting cells (see Methods).

Finally, the most direct estimates for the total amount of virus produced per infected cell was achieved using single-cycle SIV to infect PBMC which were placed back in uninfected rhesus macaques. By measuring the total amount of virus produced and accounting for clearance, this experiment yielded a total production of approximately 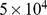 (range 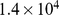
–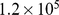) virions per infected cell [24]. Because productively infected cells have a lifespan of about one day, the cellular burst size estimates of Chen et al. [24] imply daily production rates of approximately 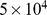 virions.

Summarizing, the latest production rate estimates converge on a few thousand to approximately 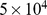 virions per productively infected cell [22]–[24]. The production rate estimates of Reilly *et al.* and Chen *et al.* depend on the viral clearance rate, 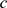. The 10-fold range in the estimated production rates is at least partly due to differences in the clearance rate used in the calculations. Reilly *et al.* [23] estimate that 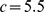 d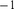 in lymphoid tissue, while Chen *et al.* [24] used a previous estimate of 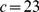 d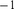 in the blood [15]. Since, our main result will be that the clearance of free virus in lymphoid tissue should be fast, and that the observed clearance from the blood is not clearance but the rate of efflux to other organs, we will vary the production rate in our analysis and consider 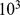 to 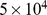 particles per cell as potential realistic estimates. Finally, note that different cell types, e.g., infected macrophages, may have different production rates than infected T cells. Here we consider that the vast majority of virus is produced by infected CD4+ T-cells [4], [25], and hence use estimates of production from those cells.

In one earlier modeling study a production rate of several thousand particles per cell was shown to be consistent with a viral half-life of 3–4 hours in the lymphoid tissue [14], suggesting that the recent estimates of 10-fold higher production rates [24] imply even shorter half-lives. However, large total viral production per infected cell [22]–[24] and the short viral half-lives they imply [14], seem difficult to reconcile with the suggestion that most of the virus in the lymphoid tissue is long-lived and in association with FDCs [5]. The problem of balancing production with clearance can be introduced by a simple calculation that assumes the body is a single well-mixed compartment. An order of magnitude estimate for the total number of productively infected cells in a human is 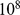 cells [5], [6]. For a human with a viral load of approximately 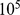 particles ml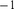 of plasma, and an estimated total of 15 liters of extracellular body water in which virus could distribute, one estimates that there are a total of 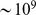 free virus particles in extracellular fluids (i.e., only about 2% of the estimated total body load) [4]. Requiring steady state in the conventional model for virus production, i.e., 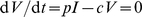, with a production rate of 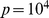 viral particles per infected cell, 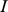, per day, and a steady state of 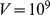 free virus particles and 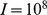 productively infected cells, one would need a *per virion* clearance rate of 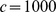 d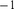, which is much higher than published estimates for the viral clearance rate in humans [15], but resembles the rapid clearance rate observed in rhesus monkeys [17]. Even if 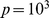 d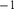 then 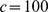 d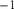 is needed to balance production, which is still larger than the current clearance rate estimate in humans [15].

In this paper we attempt to reconcile the various estimates for the viral clearance rate, the viral production rate, and the amount of long-lived virus trapped on FDCs within one modeling framework in order to test whether there is a consistent interpretation explaining all observations. To do so we introduce compartmental models to analyze recent experimental data on viral clearance in various organs, and estimate the rates at which virus is exchanged between them.

## Results

### Clearance of SIV from the plasma

A simple and direct approach to estimate the clearance rate of virus from the blood is to infuse virus particles into the blood, and monitor their disappearance by taking frequent blood samples. Zhang *et al.* [16], [17] administered large amounts of SIV to infected and uninfected rhesus macaques by an intravenous bolus injection (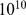 to 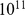 viral particles), or by constant intravenous infusion (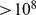 viral particles min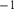). From the rate at which virus was lost from the plasma afterwards, plasma half-lives of 3–4 minutes were estimated [16], [17]. These half-lives were similar in infected and uninfected monkeys. Virus did not appear to be lost from the plasma by binding to erythrocytes, PBMCs, granulocytes, or platelets because there was no evidence of virion binding to these cell types [16]. In another experiment, viral clearance in various organs was tracked by injecting radioactively labeled SIV into macaques, and measuring the percentage of radioactivity and of SIV RNA persisting in various organs after two hours [17]. Because 30% of the radioactivity, and only 0.053% of the injected SIV RNA, was recovered from the liver (see Table 1), it was concluded that the liver plays a major role in viral degradation [17].

**Table 1:** The fraction of a bolus injection of SIV going into an organ after two hours, and the clearance rate in that organ estimated by Eqs. 3–4.

		measured		normalized		
Organ	% RNA	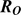	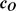 (d 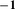 )	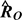	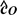 (d 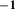 )	half life
Liver	0.053	0.295	79.75	0.728	91.28	10.9–12.5 min
Lung	0.154	0.054	44.71	0.133	56.10	17.8–22.3 min
Spleen	0.077	0.004	20.66	0.010	32.19	31.0–48.3 min
Lymph nodes^a^	1.380	0.030	9.73	0.074	21.06	47.4–102.6 min
Others^b^	0	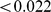	–	0.054	–	–
Total	1.664%	0.405	–	1.0	–	–

Data from Zhang *et al.* [16], [17]: for the indicated organs the fraction, 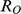, of injected radioactivity was measured in an uninfected rhesus macaque (animal AR97), and the percentage of injected SIV RNA was measured in another uninfected macaque (animal 1336). The % RNA column corresponds to the 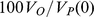 of Eq. (3). From this data we compute the clearance rate in the organ, 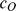, and the corresponding half-life. Because in total only 40.5% of the radioactivity was recovered, we also re-normalize the radioactivity data (assuming that part of the radioactivity has disappeared by degradation of the labeled molecules). The ranges in the half life column are obtained from the corrected and the uncorrected radioactivity data, respectively.

a Lymph nodes were assumed to be 1% of the total body weight.

b measured in heart, kidney, muscle, pancreas, brain and tonsil.

If most of the viral degradation indeed takes place in organs such as the liver [17], most of the measured clearance from the blood would be efflux from the blood into the organs. Therefore, we write the following simple model for the amount of radioactive virus in the plasma, 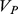, and in a particular organ, 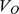,
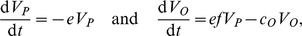
(1)where 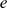 is the daily efflux from the blood, 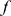 is the fraction that arrives in the particular organ, and 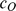 is rate of clearance in the organ. We neglect the flux from the organ back to the blood during this 2 hr experiment because this simplifies the analyses, but also because the results of the bolus injection and the continuous virus infusion experiments [16], [17], yielded similar disappearance of virus from plasma in uninfected and already infected monkeys, indicating that this flux is very small in these short-term experiments.

If 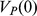 is the total amount of virus injected into the plasma, then
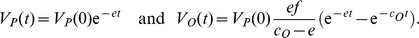
(2)After two hours, the fraction of infused viral RNA found in the organ is
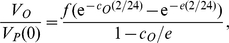
(3)where the 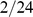 converts 2 hrs into a per day timescale. The fraction of radioactivity ending up in the organ after the two hour experiment, 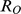, is assumed to be equal to the fraction of virus entering (and presumably degraded in) that organ, i.e.,
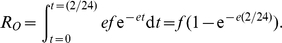
(4)


Using the estimated clearance rate from the blood of 0.2 min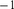 [17] as the efflux rate, 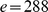 d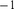, we estimate the fraction, 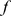, and the clearance in the organ, 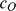, from the Zhang *et al.* [17] data shown in Table 1. Since in Eq. (4) the term 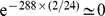, the fraction of measured radioactivity in the organ, 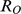, determines the parameter 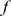 in the model. Substituting the fraction of SIV RNA in the organ, 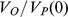, the estimated efflux, 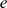, and the fraction of radioactivity, 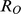, into Eq. (3), one can numerically solve for the clearance rate constants, 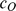, in the four organs (Table 1). The estimated clearance rates in the various organs vary from 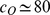 d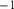 in the liver to 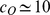 d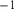 in the lymph nodes. The latter is only 2-fold faster than the clearance rate of 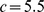 d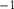 estimated by Reilly *et al.* [23] for lymphoid tissue.

Only 40.5% of the total radioactivity was recovered in the monkey 2 hr after injection. This could be due to a loss of radioactivity by viral degradation and removal of labeled molecules, or to accumulation of SIV in other body compartments, such as the gastrointestinal tract, that were not examined [17]. The former we can correct for by re-normalizing the radioactivity data so that the total is 100%. This correction doubles the estimate clearance rate in lymph nodes, and has a smaller effect on the other clearance rates (see Table 1). If the virus unaccounted for by the radioactivity data is ending up in other organs, the clearance rates based upon the uncorrected radioactivity data should be valid. Interestingly, percentages of SIV RNA were also measured in ileum, cecum, duodenum and rectum in other monkeys, and adding these data from the gut to the “Others” class hardly increased the amount of SIV RNA in that class [16]. This would argue that only a minor fraction of the injected virus ends up in the gastrointestinal tract, and/or that the clearance rate in the gastrointestinal tract is much larger than in the other organs so that SIV RNA is not found there. Unfortunately, there is no radioactivity data for the gastrointestinal tract to distinguish between these two possibilities, and we estimate the viral half-lives in the various organs by the ranges indicated in Table 1, as obtained from the corrected and the uncorrected radioactivity data, respectively. Summarizing, modeling the radioactivity data provides estimates of viral clearance rates between 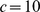 d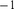 and 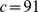 d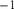 in various organs (Table 1).

### Plasma apheresis

HIV-1 clearance rates from plasma have been estimated in chronically infected patients by plasma apheresis over a period of two hours. Plasma was removed at a rate of 39 mL per min, and was replaced by an equivalent volume of isotonic saline containing 5% albumin. On average, this procedure removed a total of approximately 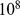 particles, and resulted in a nadir of virus equal to half the initial viral load [15]. The following model, formally equivalent to the one presented in Ramratnam *et al.* [15], was used to fit the data:
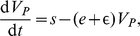
(5)where 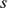 is the rate of virus influx from the lymphoid tissue, 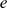 is the normal efflux from the blood, and 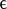 is the additional rate of virus removed from the blood due to plasma apheresis. It was assumed that over the course of the two hour experiment the flow of virus into the blood, 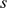, remains constant, and it was found that the rate of viral efflux in four patients ranged from 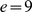 to 36 d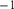, with an average of 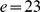 d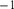 [15]. The rate of virus influx, 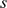, at steady state is estimated by multiplying the plasma efflux rate, 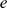, by the initial virus load, and varies from 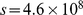 to 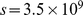 particles d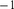, with an average of 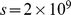 particles d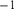. The mere fact that the removal of less than 1% of the total body virus over a period of two hours led to significant declines in the plasma virus load (Table 2 and Ramratnam *et al.* [15]), suggests that the plasma virus pool is not rapidly replenished from the lymphoid tissue or other organs[14]. This observation also supports our neglecting virus return from organs back into the plasma in Eq. (1).

**Table 2:** The plasma apheresis data from Ramratnam *et al.* [15].

Patients	1	2	3	4
Duration (min)	78	120	136	116
Total plasma volume (ml)^a^	3549.3	3709.5	4048.2	4332.9
Plasma removed (ml)	2972	2974	5319	3477
Baseline HIV load (total plasma)	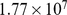	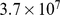	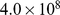	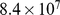
Total virus removed	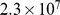	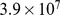	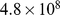	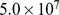
Efflux rate ( 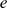 : d 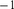 )	25.9	21.6	8.64	36.0
Extra efflux rate ( 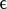 : d 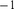 )	15.5	9.6	13.9	10.0
Total influx ( 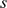 : particles d 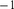 )^b^	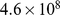	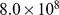	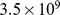	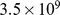
Predicted nadir	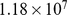	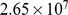	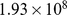	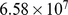

Average clearance rate from blood 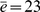 d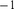 and average influx into blood 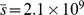 particles d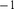.

a Estimated from body weight (44 ml/kg).

b Estimated by clearance rate 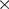 baseline HIV load (given in particles, i.e., HIV RNA/2).

It is worth noting that the estimate for the efflux rate in humans of HIV-1 from plasma is more than 10-fold slower than the estimated plasma efflux rate of SIV in rhesus monkeys [15]–[17], which could reflect a true difference between these two species. Alternatively, it could be that the plasma clearance rate in the four patients studied by Ramratnam *et al.* [15], all of which had high viral loads, is slower than in the monkeys studied which had much lower viral loads [16], [17]. A potential mechanism for the more rapid clearance in monkeys with low viral load could be the rapid attachment of virus to various receptors on blood born cells, whereas in patients with chronic high viral loads these receptors could be saturated and bind less virus (see Discussion).

### Clearance of virus from lymphoid tissue

Since most virus production takes place in the lymphoid tissue, we modify a previously published compartmental model [14] to rewrite the fixed source 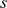 in Eq. (5) into a term depending on the amount of virus in lymphoid tissue (LT). We proceed by considering four viral compartments: virus in organs other than LT, 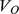, virus in the plasma, 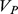, free virus in the lymphoid tissue, 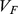, and virus bound to FDCs in lymphoid tissue, 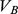. The model has two clearance rates, 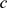 and 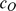, for the rate of clearance of free virus in LT, and in other organs (like the liver and lung), respectively. As before, there is no clearance in blood. Virus bound to FDCs is considered to be long-lived [7], and virus in the plasma is considered to be lost by migration to organs or LT. We allow for influx of free virus into the plasma from the LT with rate constant 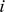, because now we are modeling a long term process, and efflux from the plasma with rate constant 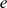. A fraction 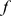 of the virus leaving the plasma will return to the LT, the rest is cleared in organs like the liver and lung. This compartment model is represented by the following equations:
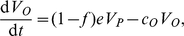
(6)

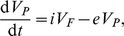
(7)

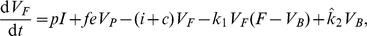
(8)

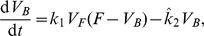
(9)where 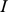 is the number of productively infected cells in the LT.

In this model we make a distinction between clearance and efflux. We speak of efflux when the number of virus particles is conserved, and speak of viral clearance in an organ only if the virus is being degraded there. Thus, these equations assume that there is no viral clearance from the blood; there only is efflux to the lymphoid tissue and to other organs that have viral clearance rates, 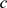 and 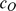, respectively. Mixing SIV with fresh blood taken from monkeys provided no evidence for viral degradation within plasma *ex vivo* [17], and for several viruses most of the clearance takes place in liver and spleen [16]. The model can easily be modified to allow for viral clearance from the blood, e.g., by decreasing the 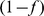 term in Eq. (6). This would not affect our results, however, because Eq. (6) represents a sink that does not affect the other three compartments of the model. Technically, the “viral clearance” rates from the blood estimated previously by the apheresis [15] and infusion [16], [17] experiments, cannot distinguish between clearance and efflux, and we will let these estimates represent the efflux rate, 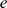, in Eq. (7).

The parameter 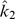 in Eqs. (8) and (9) is the average dissociation rate of virus bound to FDCs. 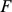 is the maximum number of binding sites on FDCs that HIV-1 can attach to, and 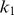 is the rate constant for the association of virus with an FDC binding site. These binding sites include complement receptors and Fc receptors [8], and possibly other receptors like DC-SIGN [26]. The dissociation process is complicated and depends on the number of bonds by which virus is bound to the FDC [8]. When 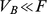 most virus particles have multiple bonds holding the virus to FDC, which makes the dissociation slow, and probably accounts for the long half-life of a fraction of the bound virus [7], [8], [11]. When 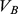 is large most viruses will have few bonds holding them to FDC and dissociation should be more rapid. We can describe this phenomenologically with a Hill-function such that the effective virion dissociation rate constant 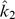 increases with 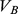


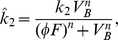
(10)where 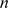 and 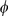 are constants with 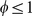, and 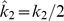 when 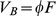.

In a typical patient the FDC pool seems saturated, i.e., 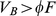, and most virus is expected to be monovalently bound with a dissociation rate estimated as 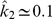/sec [8]. Image analysis combined with *in situ* hybridization suggested that most of the virus in the lymphoid tissue is associated with the FDC network [5]. The FDC associated virus, 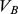, fills up early in infection [12]. During chronic infection we therefore assume 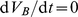, and this large pool of FDC associated virus is viewed as a filled store in quasi steady-state that contributes little to the total body viral clearance. When the FDC pool is close to being saturated, the steady state of Eqs. (6) to (9) corresponds to
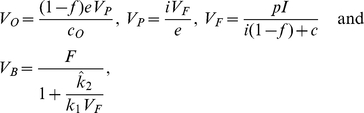
(11)where 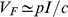 if 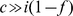, and 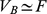 if 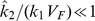.

To consider the total virus load we add Eqs. (6)–(9) yielding,
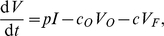
(12)where 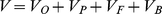 is the total body virus load. For a chronically infected patient assumed to be in a steady state we substitute 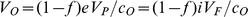 from Eq. (11) to obtain
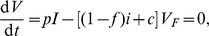
(13)where the total body clearance rate, 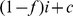, is the total steady state rate of clearance taking place in organs like the liver and the lymphoid tissues. Since our aim is to estimate 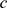, we rewrite Eq. (13) as
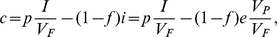
(14)where we have again used 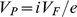 from Eq. (11).

We can use the relationships between 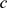 and the steady state levels in Eq. (14) to study what clearance rates would be required to balance production in a number of typical situations. Since there is ambiguity on the rate of efflux from the blood, 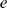, we use the efflux estimates from the plasma apheresis experiments [15], i.e., 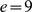 to 36 d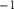, and from the rhesus monkeys [16], [17], i.e., 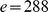 d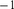, as lower and upper bounds to create examples of how viral production and clearance could be balanced in hypothetical patients. If we use a middle value for the fraction of plasma virus returning to the LT, e.g., 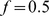, we obtain an upper estimate of 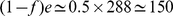 d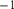 and take a lower estimate of 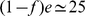 d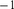. To estimate the ratios between the variables in Eq. (14) we pick an example of a patient in a chronic steady state with total body counts of 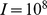 productively infected cells, 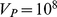 virus particles in the peripheral blood, and 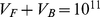 virus particles in the lymphoid tissue. Finally, because Hockett *et al.* [22] did not detect virus associated with FDCs in almost half of their patients, and measured 10-fold higher total amounts of virus in lymph nodes from those patients where they could detect virus on FDC, we consider two possibilities. To model a “typical” patient where most of the virus is associated with FDCs, we let 90% of the lymphoid tissue virus be associated with FDCs, and obtain that 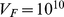. To model patients with a smaller pool of FDC associated virus, we also consider the possibility that half of the LT virus is bound to FDCs, i.e., 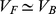, which amounts to 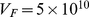. This allows us to study how the estimates for the viral clearance rate in LT depend on the fraction of virus bound to FDCs.

**Figure 1:**
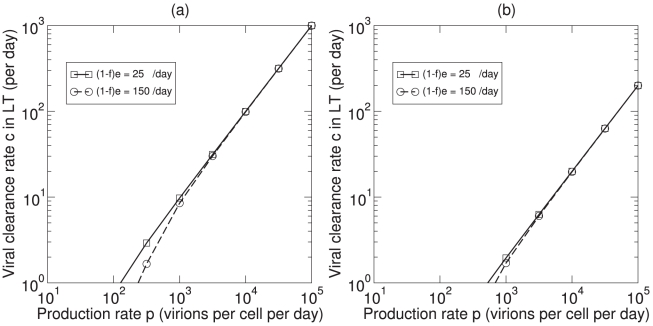
Viral clearance in the lymphoid tissue, 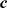, as a function of the production rate 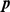, given by Eq. (14), for a typical patient with 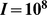 cells, 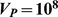, 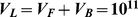 particles, and the indicated clearance rates via the blood, 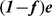. In (a) 90% of the virus in lymphoid tissue is associated with FDCs, i.e., 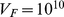, and in (b) this is 50%, i.e., 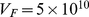.

For the more “realistic” example, Fig. 1a, where most of the virus is associated with FDCs, our estimate of the *per capita* clearance rate in LT, 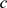, depends strongly on the production rate 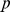, and a large production rate, e.g., 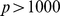 virions per cell per day, requires rapid clearance of virus in the lymphoid tissue, i.e., 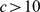 per day to maintain a steady state level of virus (Fig. 1a). The recently proposed production rates of more than 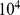 viral particles per infected CD4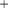 T cell would require LT clearance rates of 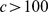 per day (Fig. 1a). When Chen *et al.* [24] estimated these high production rates they were conservatively assuming that 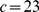 d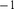. Because their estimated production is proportional to the assumed clearance rate, our new results suggest that the true production could be even higher. In cases where less virus is associated with FDCs (Fig. 1b), we find a similar relation between the clearance rate in LT, 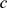, and the production rate, 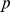, but the required clearance rate 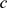 is approximately 5-fold smaller because we allow for 5-fold more free virus, i.e., 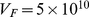 (Fig. 1b). In this case, for realistic virion production rates per cell, e.g., 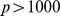 per day, the estimated clearance rate 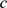 is fairly independent of rate of efflux from the blood (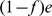); see Fig. 1.

### The viral exchange rate between lymphoid tissue and blood

Analysis of the plasma apheresis experiments in humans provided estimates for the influx of virus from the LT into the blood (Table 2). This can also be calculated from the quasi steady state of Eq. (7), i.e., for the influx of virus from the LT into the blood, one obtains that
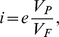
(15)where the efflux 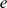 has the two estimates of 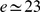 d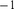 and 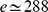 d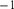 in patients and macaques, respectively. For the case when 90% of the lymphoid tissue virus is associated with FDCs, i.e., 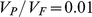, this means that 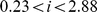 d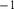. Assuming an equal distribution of free and FDC-bound virus in lymphoid tissue, i.e., 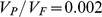, one obtains 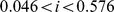 d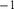. Taking these two cases as extremes, the daily influx of virus from the lymphoid tissue into the blood would be between 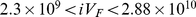 virus particles per day, i.e., about 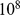 to 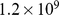 particles per hour. These estimates are in good agreement with the daily influx estimated from the plasma apheresis experiments (Table 2). Finally, for these estimates of 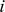, the clearance rate in LT should approach 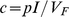 as the term 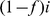 in Eq. (14) is negligible.

**Figure 2:**
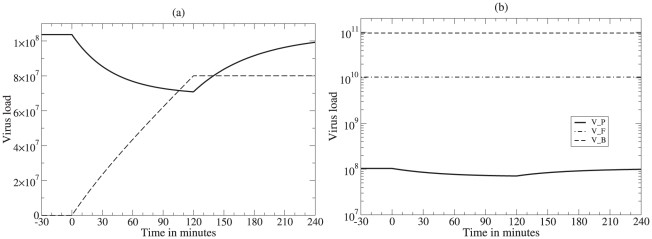
Plasma apheresis for 2 hrs simulated in the model for a patient with 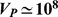, 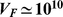 and 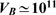 virus particles in the total body. The solid line is the plasma viral load (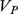). The discontinuities reflect the time period of plasma apheresis which starts at time zero and continues for two hours (during this period we set 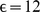 d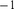; otherwise 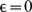). The dashed line in (a) is the total amount of virus removed (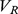), i.e., the solution of 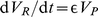. The dashed line in (b) is virus bound to FDCs (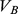), and the dash-dotted line in (b) is free virus in lymphoid tissue (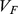). Parameters: 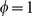, 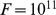, 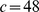 d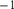, 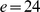 d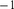, 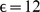 d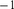, 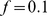, 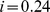 d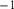, 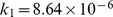 d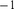, 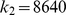 d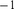 (0.1s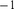), 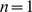, 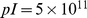 particles d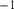. The results in this Figure are independent of the parameter 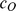 because we are only solving for 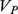, 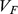, and 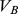 (see Eqs. 7–9).

To test whether the full model (Eqs. 6–9) is consistent with the plasma apheresis experiments, we make reasonable guesses for the other parameters of the model. Allowing rapid filling of the pool of virus bound to FDCs we set the number of FDC binding sites 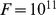 and 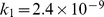 d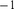. With these parameters the initial “on” rate when all FDC sites are free is 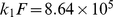 d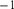 (10 s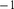). To have 100-fold more free virus in LT than in blood, i.e., 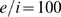 (see Eq. (11)), we set 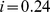 d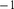 and 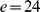 d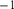. To have about 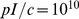 free virus particles in the LT, we set 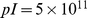 particles d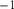 and 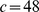 d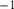 (see Eq. (11)). During a two hour plasma apheresis experiment we transiently add an estimated efflux of 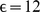 d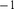 during apheresis to Eq. (7) (like we do in Eq. (5)). For these parameters the model results mimic the plasma apheresis experiments in the blood (Fig. 2a), reducing the viral load in plasma by 30% and accumulating a total of 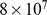 virus particles. Free and bound virus in LT are hardly affected (Fig. 2b). Very similar results are obtained when we increase production and viral clearance in the LT 10-fold to 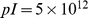 particles d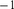 and 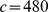 d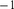 (not shown). Explaining the plasma apheresis experiments in humans therefore indeed requires a viral efflux half-life from the plasma of about half an hour [15].

Choosing the efflux rates estimated in monkeys [16], [17], we have also set 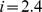 d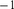 and 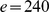 d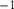, which delivers a very similar steady state as that shown at time zero in Fig. 2. Simulating plasma apheresis by setting 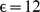 d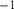 for two hours a total of 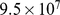 virions are removed, but the plasma virus load decreases by 5% only (not shown). This is a natural result because the additional clearance of 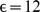 d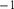 is small compared to the normal efflux from the plasma of 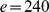 d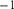. We therefore predict that if plasma apheresis experiments were repeated in SIV infected rhesus macaques, the viral load in the plasma would hardly be affected.

## Discussion

We have shown that the balance between viral production and viral clearance implies rapid *per capita* viral clearance rates in lymphoid tissues. The larger the viral production rate per productively infected cell, and the more virus that is bound to FDCs (see Fig. 1), the larger the *per capita* clearance rate of free virus in lymphoid tissue must be to balance viral production. Recent estimates of a burst size up to 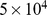 virions per cell and a productively infected cell life span of about a day [21], [24], imply viral clearance rates in the lymphoid tissue of 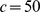 to 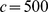 d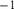 (Fig. 1).

In our modeling work the rate of virion clearance, 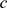, is assumed to be a constant. This is equivalent to assuming that clearance occurs by a first-order process or that virus clearance can be described by an exponential decay. However, it is possible that viral clearance obeys more complex laws. Comparing viral loads in lymph nodes and plasma from 9 patients at relatively advanced stages of disease, Hockett *et al.* [22] demonstrate that the plasma viral load increases faster than proportional with the number of productively infected cells. One possible explanation is that the viral clearance rate decreases or saturates when the viral load increases. However, this explanation remains speculative as it requires a 100-fold decrease in the clearance rate at their highest viral load, and it seems unlikely that there is such a tremendous variation in the clearance rate [22].

The efflux and/or clearance rate of SIV from the blood of uninfected monkeys and of infected monkeys with a low viral load [16], [17] is about 10-fold higher than that of HIV-1 measured by plasma apheresis experiments in chronically infected patients [15]. Because the additional removal (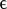 in Table 2) realized in the plasma apheresis experiments is also 10-fold smaller than these efflux rates in monkeys, plasma apheresis would have hardly any effect if humans were to have efflux rates similar to these monkeys (this expectation was confirmed by computer simulation). As discussed above this 10-fold difference in the estimated efflux rate from the plasma could reflect a true species difference. A speculative alternative is that efflux from the plasma hinges upon attachment of virus to various receptors on blood born cells, like CCR5 and CD4 on various cell types, gp340 on macrophages [27], DC-SIGN on dendritic cells [26] and DARC on red blood cells [28], [29]. Because the monkeys in these experiments had much lower viral loads than the four patients studied by apheresis [15]–[17], most of the receptors could be free in monkeys and occupied with HIV-1 in chronically infected humans with a high viral load. However, this remains speculative because Zhang *et al.* [16] found negligible amounts of virus on erythrocytes, peripheral blood mononuclear cells (PBMC), granulocytes, and platelets. Moreover, note that the estimated clearance rate in the liver, the organ that appears to be responsible for most of the peripheral viral degradation [17], is reasonably close to the high clearance rates of free virus in lymphoid tissue that we estimate to be required for balancing the total body virus production.

Finally, it may seem that the notion of a large FDC store of bound virus is incompatible with the rapid viral clearance rates seen in spleen and lymphoid tissues of uninfected macaques [16], [17]. In other words, if the FDCs were trapping and maintaining the virus in lymphoid tissues, then after radiolabeled virus was injected, the percentages of radioactivity and SIV RNA found in these tissues should have been similar, whereas a 2-fold difference was observed, i.e., 3% vs. 1.4% (Table 1). There are at least two possible explanations for this difference. First, virus is reversibly bound to FDC [8] so radiolabeled virus could dissociate and the virus could then be degraded. Alternatively, the clearance after bolus infusion of SIV in monkeys was studied over a time window of just two hours [16], [17], and one could speculate that most of the added virus in these short experiments fails to bind FDCs, and could therefore be cleared rapidly.

Rapid viral clearance in lymphoid tissue is not surprising. The lymphoid tissue contains more than 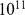 CD4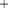 T cells, i.e., the ratio of virus to CD4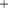 cells in the LT is approximately 1∶1, and virus particles will bind CD4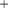 T cells and macrophages, and defective virus particles will be “cleared” by non-productive infection. Phagocytic cells that are also abundantly available in LT may clear virus via various types of receptors, like Fc, complement, and DC-SIGN on dendritic cells [26]. Since the process of virus binding cell surface receptors is relatively fast, it can readily account for the rapid clearance rates that we derive from balancing total body production with total body clearance. Finally, during chronic HIV-1 infection the long-lived pool of virus on FDCs should be in steady state, and thus not contribute to the actual clearance rate of the virus in lymphoid tissue (see Eq. (12)).

In a previous paper we showed that the rate of viral clearance in lymphoid tissue would markedly affect the estimated life span, 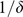, of productively infected cells deduced from antiretroviral drug therapy (ART) experiments, if the clearance rate in lymphoid tissues were sufficiently slow [14]. Since we now estimate even higher clearance rates than we did previously, it becomes even more likely that the clearance from lymphoid tissue is sufficiently fast to not affect the accuracy of current estimates of 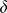, the death rate of productively infected cells. Having 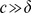, one expects the loss of productively infected cells to be the dominant slope of viral decline during the first week or two of ART [14]. The amount of virus in the blood, free virus in lymphoid tissue, and virus on FDCs should be in quasi steady state with the loss of productively infected cells, which is in good agreement with the rate of about 0.5 per day at which virus in lymphoid tissue declines during ART [13]. When the amount of virus on FDCs has dropped significantly, most of the remaining virus will be attached by multiple bonds [8], which can account for the observed long half lives of virus on FDCs during ART [7].

Summarizing, we have provided new estimates of viral efflux and clearance rates in various organs, including blood and lymphoid tissue. We have confirmed that the exchange rate from lymphoid tissue to the blood should be slow [14]. Whenever viral production rates exceed 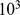 virus particles over the life time of a productively infected cell [22]–[24], we estimate clearance rates in lymphoid tissue of 10–100 d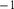 for typical situations where most of virus in lymphoid tissue is associated with FDCs.

## Methods

**Figure 3:**
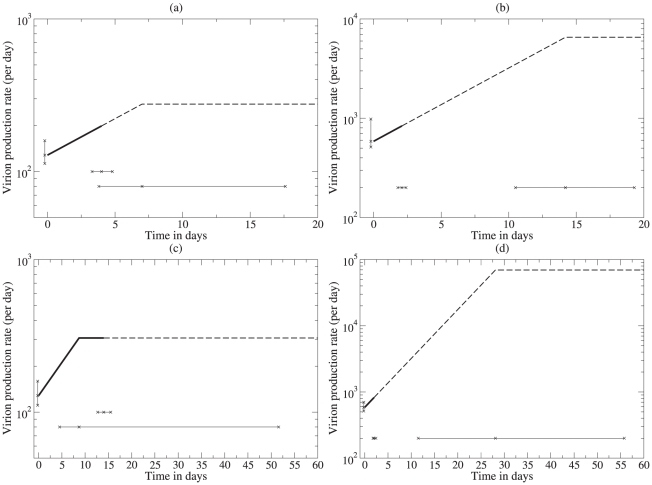
Viral production rates in the lymphoid tissue estimated by Reilly *et al.* [**23**] for resting (panels a,c) and activated cells (b,d) respectively. The top row of the Figure (Panels a,b) is the result when the mean of the prior distribution of the half-life resting cells is 4 days (and that of activated cells is 1.5 days). In the bottom row (c,d) the mean of the prior distribution of the half-life resting cells is 14 days (and that of activated cells remains 1.5 days). The median total production rate per cell is computed by integrating these curves up to the half-life of the cell (as indicated by the heavy lines) and amounts to 1479 and 1395 virions per activated cell and 644 and 3405 per resting cell (see the text). The horizontal and vertical lines marked by crosses denote the mean value with 95% credible intervals for the production rate at time zero, the half-life of the cell, and the saturation time of the production function [23].

Reilly *et al.* [23] fitted data obtained by *in situ* hybridization in lymphoid tissue, including measurements of the number SIV RNA copies on the surface of and in the vicinity of activated and resting CD4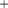 T cell in lymphoid tissue of acutely SIV-infected rhesus macaques, with averages of 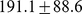 and 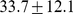 copies per cell. Although the data were static, i.e., they were single snap-shots of different cells not containing any information on the time since infection of the cells, the data was fit to a dynamic model with a Bayesian approach using simulated annealing to find the most likely parameter values [23]. The model consisted of a three parameter viral production function (see Fig. 3, i.e., intercept, up-slope, and saturation time, and two parameters for the exponential decay of cells producing virus, and loss of viral particles, respectively [23]. The prior distribution for the half-life of activated infected cells was fixed to a mean of 1.5 days, whereas having little information on the expected life span of productively infected resting cells, two different prior distributions for the half-life of resting infected cells were chosen, with means of 4 days (Fig. 3a & b) and 14 days (Fig. 3c & d), respectively.

To estimate viral production rates per virus producing cell from this data, one has to integrate the virus production function over the life span of the cells. Reilly *et al.* [23] estimated the median production rate by integrating up to the estimated half-life of the cells (see the heavy lines in Fig. 3), and this yielded median production rates of 1479 and 1395 viral particles per activated virus producing cell for the two prior distributions (Fig. 3b & d, respectively), and of 644 and 3405 viral particles per resting virus producing cell (Fig. 3a & c, respectively).

Note that the estimates obtained in Fig. 3 are independent of the estimated time to saturation, and largely depend on the intercept and slope of the virus production function (Fig. 3). In three of the four panels the heavy black line, reflecting the period over which virus production was integrated, stops well before the saturation point. This is reassuring because the 95% credible intervals on the saturation time are very large. Indeed, these data can hardly support the existence of a saturation time in the production curve because the estimated saturation time is much larger than the estimated half-life of the cells. Thus, the data must have had virtually no cells that became old enough to breach the saturation time, and therefore the data hardly contains any information on possible saturation effects. This implies that the estimated saturation times were largely determined by the prior distribution of the Bayesian parameter estimation procedure. Fortunately, for the interest of this paper, eliminating the saturation barely affects the estimated production rates.

## References

[1] Haase AT (2005). Perils at mucosal front lines for HIV and SIV and their hosts.. Nat Rev Immunol.

[2] Ho DD, Neumann AU, Perelson AS, Chen W, Leonard JM (1995). Rapid turnover of plasma virions and CD4 lymphocytes in HIV-1 infection.. Nature.

[3] Wei X, Ghosh SK, Taylor ME, Johnson VA, Emini EA (1995). Viral dynamics in human immunodeficiency virus type 1 infection.. Nature.

[4] Perelson AS, Neumann AU, Markowitz M, Leonard JM, Ho DD (1996). HIV-1 dynamics in vivo: virion clearance rate, infected cell life-span, and viral generation time.. Science.

[5] Haase AT, Henry K, Zupancic M, Sedgewick G, Faust RA (1996). Quantitative image analysis of HIV-1 infection in lymphoid tissue.. Science.

[6] Chun TW, Carruth L, Finzi D, Shen X, DiGiuseppe JA (1997). Quantification of latent tissue reservoirs and total body viral load in HIV-1 infection.. Nature.

[7] Smith BA, Gartner S, Liu Y, Perelson AS, Stilianakis NI (2001). Persistence of infectious HIV on follicular dendritic cells.. J Immunol.

[8] Hlavacek WS, Wofsy C, Perelson AS (1999). Dissociation of HIV-1 from follicular dendritic cells during HAART: mathematical analysis.. Proc Natl Acad Sci USA.

[9] Hlavacek WS, Stilianakis NI, Notermans DW, Danner SA, Perelson AS (2000). Influence of follicular dendritic cells on decay of HIV during antiretroviral therapy.. Proc Natl Acad Sci USA.

[10] Hlavacek WS, Percus JK, Percus OE, Perelson AS, Wofsy C (2002). Retention of antigen on follicular dendritic cells and B lymphocytes through complement-mediated multivalent ligand-receptor interactions: theory and application to HIV treatment.. Math Biosci.

[11] Keele BF, Tazi L, Gartner S, Liu Y, Burgon TB (2008). Characterization of the follicular dendritic cell reservoir of human immunodeficiency virus type 1.. J Virol.

[12] Schacker T, Little S, Connick E, Gebhard-Mitchell K, Zhang ZQ (2000). Rapid accumulation of human immunodeficiency virus (HIV) in lymphatic tissue reservoirs during acute and early HIV infection: implications for timing of antiretroviral therapy.. J Infect Dis.

[13] Zhang ZQ, Notermans DW, Sedgewick G, Cavert W, Wietgrefe S (1998). Kinetics of CD4^+^ T cell repopulation of lymphoid tissues after treatment of HIV-1 infection.. Proc Natl Acad Sci USA.

[14] Müller V, Marée AFM, De Boer RJ (2001). Release of virus from lymphoid tissue affects human immunodeficiency virus type 1 and hepatitis C virus kinetics in the blood.. J Virol.

[15] Ramratnam B, Bonhoeffer S, Binley J, Hurley A, Zhang L (1999). Rapid production and clearance of HIV-1 and hepatitis C virus assessed by large volume plasma apheresis.. Lancet.

[16] Zhang L, Dailey PJ, He T, Gettie A, Bonhoeffer S (1999). Rapid clearance of simian immunodeficiency virus particles from plasma of rhesus macaques.. J Virol.

[17] Zhang L, Dailey PJ, Gettie A, Blanchard J, Ho DD (2002). The liver is a major organ for clearing simian immunodeficiency virus in rhesus monkeys.. J Virol.

[18] Dumaurier MJ, Gratton S, Wain-Hobson S, Cheynier R (2005). The majority of human immunodeficiency virus type 1 particles present within splenic germinal centres are produced locally.. J Gen Virol.

[19] Haase AT, Stowring L, Harris JD, Traynor B, Ventura P (1982). Visna DNA synthesis and the tempo of infection in vitro.. Virology.

[20] Nelson PW, Gilchrist MA, Coombs D, Hyman JM, Perelson AS (2004). An age-structured model of hiv infection that allows for variations in the production rate of viral particles and the death rate of productively infected cells.. Math Biosci Eng.

[21] Markowitz M, Louie M, Hurley A, Sun E, Di Mascio M (2003). A novel antiviral intervention results in more accurate assessment of human immunodeficiency virus type 1 replication dynamics and T-cell decay in vivo.. J Virol.

[22] Hockett RD, Michael Kilby J, Derdeyn CA, Saag MS, Sillers M (1999). Constant mean viral copy number per infected cell in tissues regardless of high, low, or undetectable plasma HIV RNA.. J Exp Med.

[23] Reilly C, Wietgrefe S, Sedgewick G, Haase A (2007). Determination of simian immunodeficiency virus production by infected activated and resting cells.. AIDS.

[24] Chen HY, Di Mascio M, Perelson AS, Ho DD, Zhang L (2007). Determination of virus burst size in vivo using a single-cycle SIV in rhesus macaques.. Proc Natl Acad Sci USA.

[25] Perelson AS, Essunger P, Cao Y, Vesanen M, Hurley A (1997). Decay characteristics of HIV-1-infected compartments during combination therapy.. Nature.

[26] Geijtenbeek TB, Kwon DS, Torensma R, Van Vliet SJ, Van Duijnhoven GC (2000). DC-SIGN, a dendritic cell-specific HIV-1-binding protein that enhances trans-infection of T cells.. Cell.

[27] Cannon G, Yi Y, Ni H, Stoddard E, Scales DA (2008). HIV envelope binding by macrophage-expressed gp340 promotes HIV-1 infection.. J Immunol.

[28] Lachgar A, Jaureguiberry G, Le Buenac H, Bizzini B, Zagury JF (1998). Binding of HIV-1 to RBCs involves the Duffy antigen receptors for chemokines (DARC).. Biomed Pharmacother.

[29] He W, Neil S, Kulkarni H, Wright E, Agan BK (2008). Duffy antigen receptor for chemokines mediates trans-infection of HIV-1 from red blood cells to target cells and affects HIV-AIDS susceptibility.. Cell Host Microbe.

